# Clinical outcomes for ibrutinib in relapsed or refractory mantle cell lymphoma in real‐world experience

**DOI:** 10.1002/cam4.2565

**Published:** 2019-09-27

**Authors:** Young‐Woo Jeon, Seugyun Yoon, Gi June Min, Sung‐Soo Park, Silvia Park, Jae‐Ho Yoon, Sung‐Eun Lee, Byung‐Sik Cho, Ki‐Seong Eom, Yoo‐Jin Kim, Hee‐Je Kim, Seok Lee, Chang‐Ki Min, Jong Wook Lee, Seok‐Goo Cho

**Affiliations:** ^1^ Division of Lymphoma‐Myeloma Catholic Hematology Hospital Seoul St. Mary's Hospital College of Medicine The Catholic University of Korea Seoul Korea; ^2^ Institute for Translational Research and Molecular Imaging Catholic Institutes of Medical Science Seoul Korea; ^3^ Laboratory of Immune Regulation Convergent Research Consortium for Immunologic Disease Seoul St. Mary's Hospital Seoul Korea

**Keywords:** allogeneic stem transplantation, bendamustine, ibrutinib, mantle cell lymphoma, nonresponder

## Abstract

Ibrutinib is highly effective in patients with relapsed or refractory mantle cell lymphoma (MCL) in major clinical trials. Although there has been a dramatic improvement in survival outcomes in the salvage setting, nonresponders to ibrutinib have a bleak prognosis. Therefore, this retrospective study was conducted to identify the most appropriate therapeutic strategy and prognosis‐related factors to predict the response of patients with relapsed or refractory MCL to ibrutinib monotherapy. Thirty‐three consecutive refractory or relapsed MCL patients treated with ibrutinib were analyzed in this study. The median overall survival (OS) and progression‐free survival (PFS) after initiation of ibrutinib were 35.1 months and 27.4 months, respectively. Risk factor analysis showed that high risk according to the Mantle Cell Lymphoma International Prognostic Index (MIPI) and nonresponse to ibrutinib at the first three cycles were significantly associated with inferior OS. Poor PFS was associated with high‐risk biologic MIPI, prior bendamustine exposure, and nonresponse to ibrutinib during the first three cycles. After ibrutinib failure, primary nonresponders had poorer OS and PFS than inconsistent responders. The overall response rate for the first salvage therapy was only 33%, with a median TTP of 3.2 months. There was no effective therapeutic strategy except for allogeneic hematopoietic stem cell transplantation (allo‐HSCT). Although ibrutinib responders exhibited favorable survival outcomes, nonresponders had a dismal prognosis. To overcome these limitations, it may be necessary to modify therapeutic strategies, such as selecting inconsistent responders for earlier allo‐HSCT.

## Graphical abstract

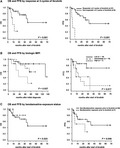

## 1 INTRODUCTION

Mantle cell lymphoma (MCL) is a rare form of B‐cell non‐Hodgkin lymphoma (NHL) that accounts for 6%‐8% of all NHLs.^1^ It affects more males than females (3:1 ratio), with a median age ranging between 65 and 68 years at initial diagnosis.^1^, ^2^ Although many patients with MCL initially respond well to therapy, most ultimately relapse and progress to disseminated refractory lymphoma with a remission duration as short as up to 3 years with median overall survival (OS) of 4‐5 years.^3^, ^4^ Given the nature of these disease characteristics, maximizing the effect of salvage therapy for patients with relapsed MCL is an important therapeutic strategy.

In the relapsed or refractory setting, advanced understanding of basic MCL‐related biology has identified potential novel therapeutic agents. Among them, ibrutinib, a first‐in‐class Bruton's tyrosine kinase (BTK) inhibitor, inhibits B‐cell receptor signaling within malignant B cells to mitigate downstream cell growth, proliferation, survival, adhesion, and migration.^5^, ^6^

Three representative ibrutinib studies enrolled patients with relapsed or refractory MCL. A phase 2 trial by Wang et al (PCYC‐1104) is the primary research that definitively influenced the general use of this drug in patients with relapsed or refractory MCL. Oral ibrutinib at a daily dose of 560 mg had durable efficacy in 111 patients with relapsed or refractory MCL (median progression‐free survival [PFS], 13.9 months; overall response rate [ORR], 68%).^7^ In updated long‐term follow‐up data, the study showed a continued duration of response (DOR) (17.5 months at 2 years).^8^ Also, recent long‐term follow‐up data from the RAY study, which is a randomized, open‐label study comparing ibrutinib and temsirolimus, confirmed significantly improved ORR and PFS in the ibrutinib therapy group compared with temsirolimus.^9^, ^10^

Based on these novel clinical studies and increasing amounts of positive objective evidence indicating the efficiency and safety of ibrutinib, it is emerging as the preferred standard therapeutic strategy in current clinical guidelines for relapsed or refractory disease.^11^ Despite these significant advances, approximately one‐third of all ibrutinib‐treated patients are nonresponders. Also, primary resistance or loss of response to ibrutinib has been sporadically reported in real‐world practice.^12^, ^13^, ^14^ Several studies have investigated ibrutinib resistance, and various resistance mechanisms have been hypothesized, including BTK binding site mutations.^15^, ^16^

However, these multicenter prospective studies generally had focused on the therapeutic response of ibrutinib, so there is less interest in the survival outcome such as OS or PFS in clinical practice. In addition, the limited data are available regarding the impact of therapeutic strategy and prognostic factors on predicting the ibrutinib response. Therefore, we conducted the current study to understand the clinical outcomes of ibrutinib therapy in the salvage setting with the same therapeutic protocol in a single center, and to identify the most appropriate therapeutic strategy for patients with relapsed or refractory MCL in the ibrutinib era.

## 2 PATIENTS AND METHODS

### 2.1 Patients

A retrospective observational cohort study was planned to assess the effectiveness and prognostic implications of ibrutinib salvage monotherapy. Consecutive adult patients who were diagnosed with histopathologically confirmed CD20‐positive, cyclin D1‐positive MCL according to the current World Health Organization classification^17^ were screened from January 2013 to August 2018 at a single center (Lymphoma‐Myeloma division, Catholic Hematology Hospital, Seoul). Among them, individuals with a relapsed or refractory disease status who received salvage therapy with ibrutinib monotherapy were included in the study. Patients who were undergoing non‐ibrutinib therapies followed by ibrutinib maintenance were excluded. A “consistent responder” was defined as achieving complete remission (CR) or a partial response (PR) from the start of ibrutinib monotherapy until the last follow‐up, an “inconsistent responder” had a loss of therapeutic response (progressive disease [PD] after achieving CR or PR with ibrutinib), and a “primary nonresponder” had stable disease (SD) or PD after the first three cycles of beginning ibrutinib. Based on the response status to ibrutinib therapy, we considered “inconsistent responders” and “primary nonresponders” to be ibrutinib failures. Clinical data, including demographic information, initial or salvage chemotherapy, and response to initial or salvage chemotherapy, were extracted retrospectively from electronic medical records. The study protocol was approved by the institutional review board of Seoul St. Mary's Hospital of The Catholic University of Korea and was in accordance with the Declaration of Helsinki.

### 2.2 Therapeutic strategy and clinical therapeutic response evaluation

Patients were administered fixed continuous doses of oral ibrutinib 560 mg per day until disease progression or unacceptable toxicity. Ibrutinib therapeutic dosing was withheld for any grade ≥3 nonhematologic toxicity, and then treatment was restarted after complete resolution or improvement within two weeks. Response to therapy was assessed after every three or four cycles of ibrutinib monotherapy. Assessments included a physical examination, blood counts, a serum chemistry profile, and computed tomography (CT) scans. Ki‐67 index was determined according to consensus criteria.^18^ While bone marrow (BM) aspiration with biopsy was performed mandatorily at initial diagnosis, repeated BM aspirations were performed on selected patients with persistent pancytopenia or peripheral blast cells in a refractory or relapsed setting. When SD or PD was confirmed, ibrutinib administration was discontinued. Response criteria were defined according to the Lugano Classification^19^, ^20^: CR was defined as the absence of a palpable mass, a normalized size on CT scan, and a negative FDG‐PET scan, without the appearance of new lesions for at least 4 weeks. PR required at least a 50% reduction in the size of the measurable lymphoma mass on CT without the appearance of a new lesion for at least 4 weeks. SD was defined as no reduction in assessable lymphoma. PD was defined as the appearance of new lesions or a ≥25% increase in tumor volume. Relapse was defined as a new disease in patients with CR or PR. We conducted a response evaluation using CT every 3 months and a response assessment using PET CT every 6 months.

### 2.3 Statistical analysis

PFS was defined as the day from ibrutinib monotherapy to the time of documented disease progression, disease recurrence, or death. OS was calculated from the time of initiation of ibrutinib administration to any cause of death. Patients with documented disease progression or death or who were lost to follow‐up were censored from further analysis.

Surviving patients were censored on the last day of follow‐up. All ibrutinib monotherapy‐related categorical variables are expressed as proportions and were compared using chi‐squared and Fisher's exact tests. Continuous variables are expressed as medians with ranges, and two groups were compared using Mann‐Whitney U‐tests. All patients were classified into one of four types of Mantle Cell Lymphoma International Prognostic Index (MIPI) scores using the International Prognostic Index (IPI) as follows: standard MIPI, simplified MIPI, biologic MIPI, and combined MIPI.^21^ PFS and OS were calculated using the Kaplan‐Meier survival method with log‐rank analysis. Cox regression was used to perform univariate and multivariate analyses to assess the independent impact of various factors on PFS and OS. Cumulative incidence estimates of relapse were calculated according to relapse or death from other causes defined as competitive events using Gray tests for univariate analysis and the Fine‐Gray method for proportional hazards regressions. All statistical analyses were performed using R version 3.2.0 (Comprehensive R Archive Network project, http://cran.us.r-project.org) with EZR graphical user interface by Kanda (Saitama Medical Center, Jichi Medical University).^22^

## 3 RESULTS

### 3.1 Clinical characteristics of all ibrutinib‐treated patients

**Figure 1:**
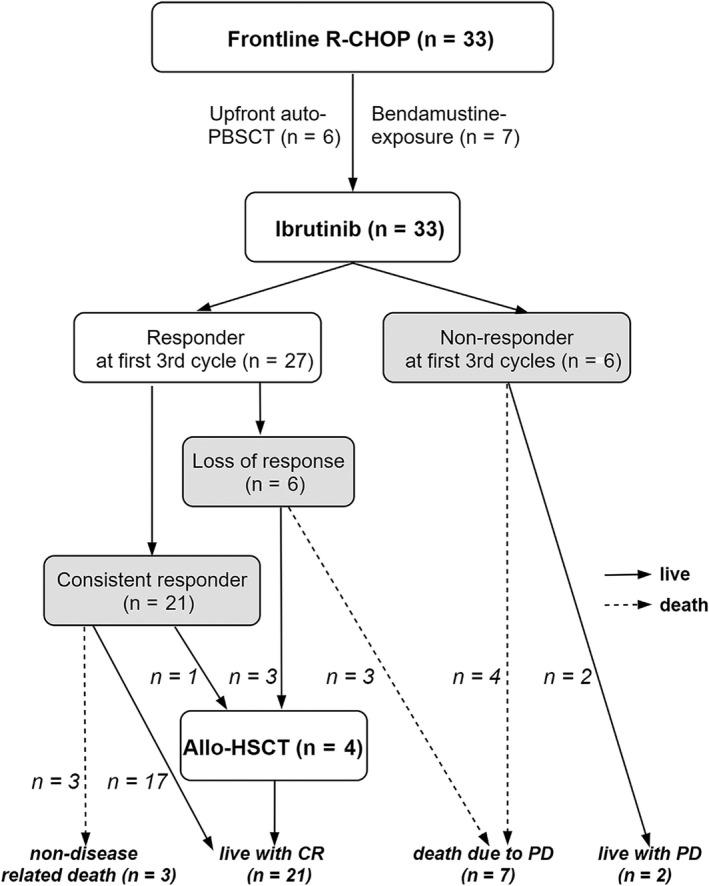
Therapeutic scheme of 33 mantle cell lymphoma (MCL) patients treated with ibrutinib

Thirty‐three consecutive patients were treated with ibrutinib monotherapy in a relapsed or refractory setting. Among them, 20 consistent responders were still receiving treatment, but one patient had electively stopped ibrutinib due to remission after receiving allo‐HSCT. Six inconsistent responders and six primary nonresponders had discontinued ibrutinib therapy. The clinical baseline characteristics before the start of ibrutinib, including staging and the MCL‐specific risk classification system are provided in Table 1, and the therapeutic sequences and results are shown in Figure 1. The median age at diagnosis was 65 years (range, 40‐79 years) and most patients had a tolerable Eastern Cooperative Oncology Group (ECOG) performance status (score 0‐1; n = 29, 87.9%); the population was predominantly male (male:female ratio, 4:1). Approximately half of the patients (57.6%) were diagnosed with BM involvement with malignant cells. According to the MCL‐related scoring system, most patients had advanced disease (84.9%, Ann Arbor stage III–IV; 57.6%, standard or simplified MIPI, intermediate to high risk; 81.8% biologic MIPI intermediate to high risk; 36.4% biologic MIPI high‐intermediate to high risk). Ibrutinib monotherapy was administered early to the patients during the MCL therapy; 69.7% of patients received one or two different types of chemotherapy before treatment with ibrutinib. All of the patients were treated with R‐CHOP regimen as first‐line chemotherapy. Among them, only six patients were treated with upfront auto‐HSCT, and seven patients were treated with Bendamustine and Rituximab combination chemotherapy (BR). There were no patients receiving Rituximab maintenance monotherapy after conventional chemotherapy.

**Table 1:** Patient characteristics and demographics at baseline

Factors	Ibrutinib responder (n = 21), No. (%)	Ibrutinib failure (n = 12), No. (%)	Total (n = 33), No. (%)	*P*‐value
Gender				.522
Male	16 (76.2)	11 (91.7)	27 (81.8)	
Female	5 (23.8)	1 (8.3)	6 (18.2)	
Age at diagnosis, median (range)	65 (40‐78)	67 (46‐79)	65 (40‐79)	.587
LDH, U/L (range)	387 (255‐928)	452 (277‐980)	417 (255‐980)	.340
WBC /10^9^ (range)	6.2 (2.3‐13.7)	7.8 (4.6‐10.8)	6.9 (2.3‐13.7)	.096
Beta2 microglobulin, µg/mL	2.4 (1.4‐22.4)	4.1 (2.2‐6.5)	2.9 (1.4‐22.4)	.002
B symptom, yes	9 (42.9)	5 (41.7)	14 (42.4)	1.000
No. of extranodal nodes involved				.363
0‐2	10 (47.6)	3 (25.0)	13 (39.4)	
≥3	11 (52.4)	9 (75.0)	20 (60.6)	
Bulky mass (≥7 cm), yes	1 (4.8)	2 (16.7)	3 (9.1)	.607
Ki‐67 index, elevated (≥30%)	6 (28.6)	5 (41.7)	11 (33.3)	.701
BM involvement, yes	11 (52.4)	8 (66.7)	19 (57.6)	.665
Chromosomal abnormality, yes	2 (9.5)	2 (16.7)	4 (12.1)	.960
ECOG performance				.003
0‐1	21 (100)	8 (66.7)	29 (87.9)	
2‐3	0	4 (33.3)	4 (12.1)	
Staging system at initial diagnosis				
Ann‐Arbor stage				.979
II	3 (14.3)	2 (16.7)	5 (15.2)	
III	2 (9.5)	1 (8.3)	3 (9.1)	
IV	16 (76.2)	9 (75.0)	25 (75.8)	
Standard MIPI				.242
Low	11 (52.4)	3 (25.0)	14 (42.4)	
Intermediate	6 (28.6)	4 (33.3)	10 (30.3)	
High	4 (19.0)	5 (41.7)	9 (27.3)	
Simplified MIPI				.087
Low	13 (61.9)	1 (8.3)	14 (42.4)	
Intermediate	6 (28.6)	6 (50.0)	12 (36.4)	
High	2 (9.5)	5 (41.7)	7 (21.2)	
Biologic MIPI				.461
Low	5 (23.8)	1 (8.3)	6 (18.2)	
Intermediate	6 (28.6)	3 (25.0)	9 (27.3)	
High	10 (47.6)	8 (66.7)	18 (54.5)	
Combined MIPI				.125
Low	6 (28.6)	2 (16.7)	8 (24.2)	
Low‐intermediate	10 (47.6)	3 (25.0)	13 (39.4)	
High‐intermediate	5 (23.8)	5 (41.7)	10 (30.3)	
High	0	2 (16.7)	2 (6.1)	
Lines of previous chemotherapy				.474
1	8 (38.1)	3 (25.0)	11 (33.3)	
2	6 (28.6)	6 (50.0)	12 (36.4)	
3	5 (23.8)	3 (25.0)	8 (24.2)	
4	2 (9.5)	0	2 (6.1)	
Prior auto‐HSCT	3 (14.3)	3 (25.0)	6 (18.2)	.691
Prior allo‐HSCT	0	0	0	
Prior bendamustine exposure	2 (9.5)	5 (41.7)	7 (21.2)	.059
Response to ibrutinib				.002
CR	18 (85.7)	3 (25.0)	21 (63.6)	
PR	3 (14.3)	3 (25.0)	6 (18.2)	
SD	0	1 (8.3)	1 (3.0)	
PD	0	5 (41.7)	5 (15.2)	
Median duration of ibrutinib therapy, months	15 (3‐69)	17 (3‐34)	16 (3‐69)	.272

Patients were classified into two groups according to ibrutinib response patterns: ibrutinib responders (consistent responders to ibrutinib, n = 21) or ibrutinib failure (inconsistent responders or primary nonresponders to ibrutinib, n = 12). There were no differences in clinical characteristics such as MCL‐specific risk classification system and duration of ibrutinib therapy between the two groups, except for *β*2‐microglobulin levels (2.4 vs 4.1 µg/mL, *P* = .002) and poor ECOG performance status (ECOG score 2‐3, 0% vs 33%, *P* = .003).

### 3.2 General clinical outcomes after salvage ibrutinib monotherapy

For all patients included in the analysis, the ORR after the first three cycles of ibrutinib therapy was 82% (n = 27), which consisted of 18% CR (n = 6) and 64% PR (n = 21). However, the final ORR was 64% (n = 21) which consisted of 15% CR (n = 5) and 48% PR (n = 16); six patients (18%) lost their initial response to ibrutinib (three with initial CR and three with initial PR). The remaining six patients (18%) did not respond to ibrutinib after three cycles (one patient had stable disease [3%] and five patients had progressive disease [15%]). Therefore, six patients (18%) were primarily refractory to ibrutinib.

**Figure 2:**
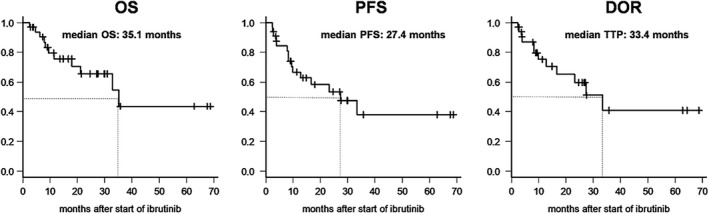
Overall survival of patients receiving ibrutinib therapy for refractory or relapsed MCL. The median overall survival (OS), progression‐free survival (PFS), and duration of response (DOR) were 35.1, 27.4, and 33.4 months after the start of ibrutinib treatment, respectively

The median OS, PFS, and DOR after initiation of ibrutinib were 35.1, 27.4, and 33.4 months, respectively (Figure 2). The median duration of ibrutinib therapy was 16 months (range, 3‐69 months) for the entire cohort. The main cause of ibrutinib discontinuation was disease progression upon treatment (and one elective discontinuation for allo‐HSCT), documented in 12 patients (36.4%), with primary refractoriness to ibrutinib in six patients (18.2%) and disease progression after initial CR or PR in six patients (18.2%). Three patients discontinued ibrutinib owing to drug‐related complications, community‐acquired pneumonia with septic shock (n = 2, 6.1%), and acute myocardial infarction (n = 1, 3.0%). Also, two patients had ibrutinib‐related atrial fibrillation (n = 2, 6.1%); one of the two was withheld from ibrutinib therapy due to the progression of acute myocardial infarction finally (this patient was progressed to acute myocardial infarction in the above mentioned), and the other one was manageable with cardiac medications and kept ibrutinib therapy without interruption. Of the patients who discontinued ibrutinib due to drug‐associated toxicities, all subsequently experienced disease progression and died. There were no discontinuations due to medication compliance problems and no bleeding‐related adverse events.

### 3.3 Prognostic factor analysis for survival outcomes during ibrutinib therapy

Prognostic factor analysis for OS or PFS showed that an advanced MCL‐related classification system rating (high‐intermediate‐ to high‐risk IPI, high‐risk standard MIPI, high‐risk simplified MIPI, high‐risk biologic MIPI, and high‐intermediate‐ to high‐risk combined MIPI) nonresponse to ibrutinib at the first three cycles and nonresponse to ibrutinib during ongoing treatment were significantly adversely prognostic according to univariate analysis (Table S1).

**Figure 3:**
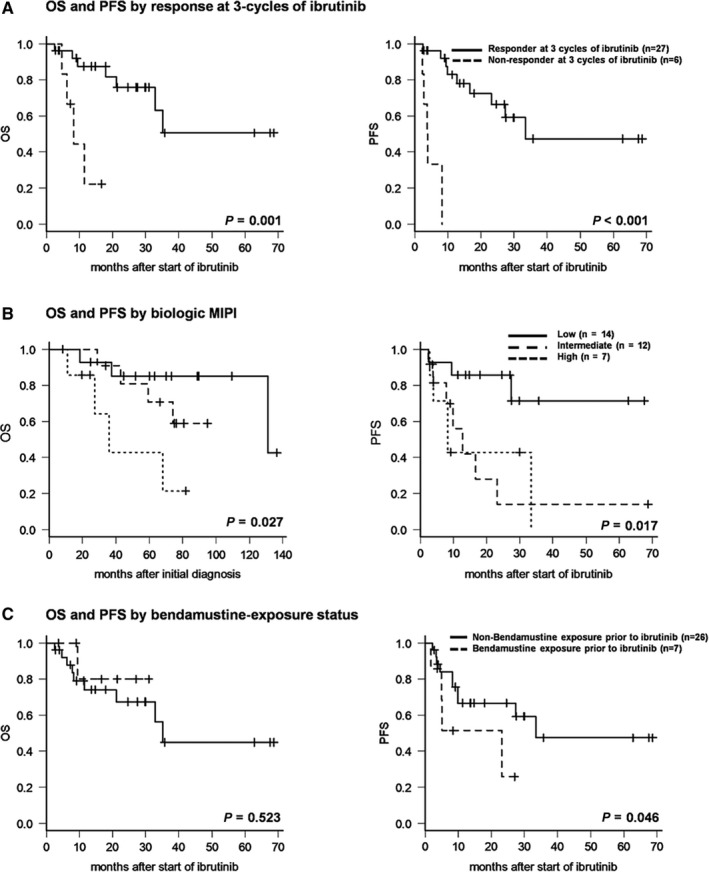
Survival outcomes according to prognostic factors. Overall survival (OS) and progression‐free survival (PFS) according to therapeutic response at first three cycles of ibrutinib (A), biologic MIPI (B), and status of bendamustine exposure (C)

Each MCL‐associated classification system was a significant independent factor in the univariate analysis. However, to exclude the effects of interference between these factors, only biologic MIPI system, which had the highest statistical significance among the MCL‐associated classification systems, was used for multivariate analysis. As a result, high‐risk biologic MIPI (hazard ratio [HR], 12.74; 95% confidence interval [CI], 1.58‐102.5; *P* = .017) and nonresponse to ibrutinib at the first three cycles (HR, 5.57; 95% CI, 1.20‐25.92; *P* = .029) were significantly associated with inferior OS (Figure 3A,B). Poor PFS was associated with high‐risk biologic MIPI (HR, 5.47; 95% CI,1.24‐24.22; *P* = .025), prior bendamustine exposure (HR, 6.65; 95% CI, 1.36‐32.56, *P* = .019), and nonresponse to ibrutinib at the first three cycles (HR, 29.97; 95% CI, 4.80‐197.2; *P* < .0001) (Table 2, Figure 3A‐C).

**Table 2:** Multivariate analysis of predictive prognostic factors affecting ibrutinib response

Factors	OS		PFS	
	HR (95% CI)	*P*‐value	HR (95% CI)	*P*‐value
Prior bendamustine‐based therapy	—	—		
No			1	1.36‐32.56
Yes			6.65	.019
Biologic MIPI				
Low to intermediate	1	1.58‐102.5	1	1.24‐24.22
High	12.74	.017	5.47	.025
Response to ibrutinib at first three cycles				
Sensitive (CR or PR)	1	1.20‐25.92	1	4.80‐187.2
Refractory (SD or PD)	5.57	.029	29.97	<.0001
Ongoing (overall) response to ibrutinib			—	—
Sensitive (CR or PR)	1	.40‐8.72		
Refractory (SD or PD)	1.87	.424		

### 3.4 Clinical outcomes and post‐ibrutinib therapy in the ibrutinib‐failure group

**Figure 4:**
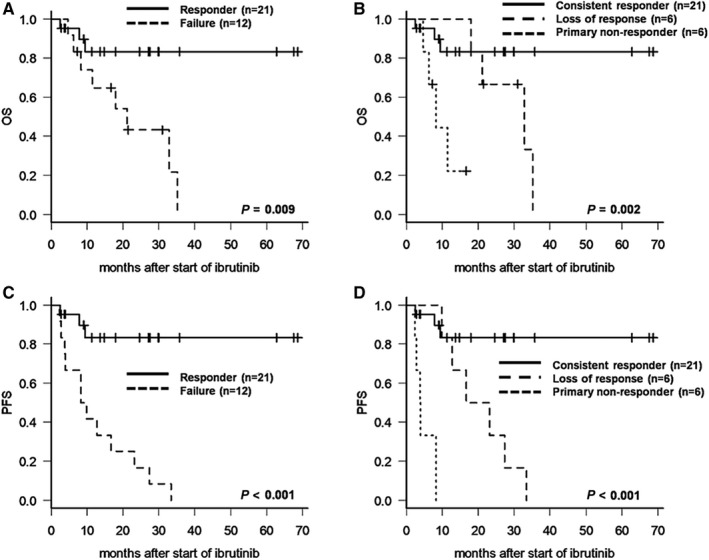
Survival outcomes according to subgroup classification for responsiveness to ibrutinib (A) Overall survival (OS) differed between ibrutinib responders and nonresponders. (B) After stratification by responsiveness to ibrutinib, primary nonresponders had an inferior OS compared with loss‐of‐response or consistent responders. (C) Progression‐free survival (PFS) differed between responders and treatment failures. (D) Subgroup analysis by responsiveness to ibrutinib showed that primary nonresponders had more inferior PFS than loss‐of‐response or consistent responders

In the subgroup analysis of the ibrutinib failure group (n = 12), the median duration of ibrutinib treatment was 17 months (range, 3‐34 months). Seven patients (58%) died after ibrutinib refractoriness. The median OS after the start of ibrutinib (Figure 4A) and the median OS after the time at ibrutinib resistance were 21 months and 4.6 months (range, 1.8‐30.4 months), respectively. The median PFS after ibrutinib therapy was 8.3 months in the ibrutinib failure group (Figure 4C). In addition, the median PFS in subgroup analysis according to primary refractory group and loss of response was 0.4 months and 19.2 months, respectively (Figure 4D). Unlike the ibrutinib failure group, only three patients in the ibrutinib responder group died due to nondisease‐related causes (14%), and ibrutinib‐related median OS and PFS were not reached (Figure 4A,B).

Among the 12 ibrutinib failure cases, only six patients (50%) received salvage chemotherapy after ibrutinib resistance. Six patients (three inconsistent responders and three primary nonresponders) were not treated with subsequent chemotherapy as they all died shortly after disease progression. The salvage chemotherapies were mainly conventional regimens, including BR (n = 3); etoposide, methylprednisolone, high‐dose Ara‐C, and Platinol (ESHAP) (n = 2); and dexamethasone, high‐dose Ara‐C, and Platinol (DHAP) (n = 1). The ORR for the first salvage chemotherapy after ibrutinib failure was 33% (two of six patients) with a median DOR of 3.2 months (range, 1.8‐6.1 months). Three patients underwent allo‐HSCT (two after achieving a response to salvage chemotherapy and one at refractory disease status), and all remained alive and in remission for at least 12 months after transplant.

Among the risk factors related to therapeutic strategy, prior bendamustine‐based therapy exhibited inferior PFS (HR, 2.507; 95% CI, 0.75‐8.41; *P* = .048) on univariate analysis (Table S1) and the survival curve for PFS (Figure 4A), although useful independent significance disappeared with multivariate analysis (HR, 3.24; 95% CI, 0.93‐11.31; *P* = .066). However, there was no significant difference in OS between patients with and without prior bendamustine exposure (HR, 1.64; 95% CI 0.73‐3.67; *P* = .228) (Table S1).

### 3.5 HSCT and ibrutinib

In MCL patients undergoing ibrutinib therapy, six patients received upfront auto‐HSCT (18.2%) before ibrutinib therapy, and no patients had auto‐HSCT after becoming refractory to ibrutinib. In cases receiving allo‐HSCT, three underwent allo‐HSCT after ibrutinib treatment failed and one patient received allo‐HSCT as a bridging therapy with ibrutinib; all of these patients were in the auto‐HSCT failure group. No patients received allo‐HSCT before ibrutinib treatment. Three patients who underwent allo‐HSCT survived and maintained a disease‐free status for at least 12 months.

## 4 DISCUSSION

Although large cohort studies in MCL patients treated with ibrutinib are rare, the prognosis of patients with refractoriness to ibrutinib is very poor.^23^, ^24^, ^25^ In this retrospective study performed at a single center, we evaluated relapsed or refractory MCL patients treated with ibrutinib monotherapy in routine clinical practice. This report confirms that MCL patients with ibrutinib refractoriness had very poor survival prognoses in the salvage setting. First, approximately 80% of ibrutinib‐treated patients had favorable responses during early ibrutinib administration. However, roughly 20% of early responders had a response duration that was not prolonged, and the patients with a loss of response had a similar dismal prognosis to primary nonresponders. In our cohort, no baseline biological factors predicted the survival outcomes associated with ibrutinib responses in multivariate analysis. However, some overall risk factors were linked with significant survival outcomes: inferior OS was related to a high risk of biologic MIPI at initial diagnosis and a lack of response to ibrutinib during the first three cycles. Also, poor PFS was associated with high‐risk biologic MIPI, early refractoriness to ibrutinib, and prior bendamustine exposure.

In an analysis of survival outcomes, previous reference trials reported similar results: PCYC‐1104^7^ and RAY^9^ reported ORRs of 68% and 72%, a median OS of 22.5 months and not reached, and a median PFS of 13.9 months and 14.6 months with 27 months and 20 months of median follow‐up duration, respectively. In our analysis of 33 patients, the ORR at the early period (82%) was higher than in previous studies, but was similar to the aforementioned studies when only patients with a consistent response were analyzed (ORR, 64% [21/33 patients]). Risk factor analyses to predict survival outcomes with ibrutinib therapy were performed in several studies. Although the results may vary, the major poor prognostic factors were a higher risk of MIPI and ibrutinib failure.^23^ As expected, multivariate analysis revealed that high risk of biologic MIPI and primary refractoriness to ibrutinib were poor prognostic factors. Inconsistent responders had improved survival outcomes initially, but eventually had poor survival outcomes. This is an indirect reminder that therapeutic strategies to maximize the efficacy of ibrutinib are needed in the salvage setting.

**Figure 5:**
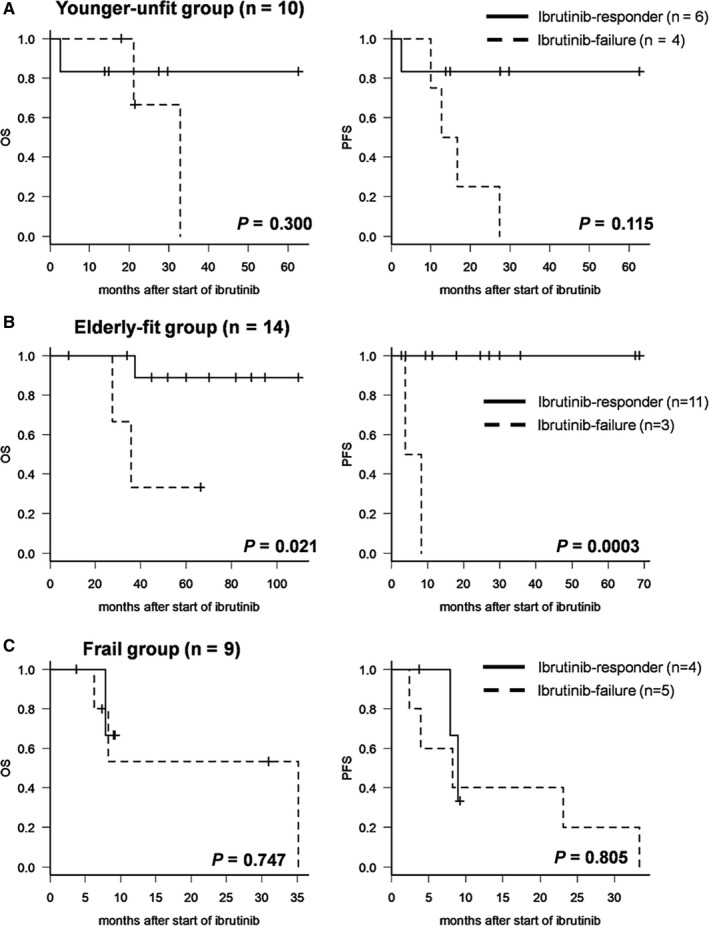
Survival outcomes according to subgroup of young, elderly, and frail patient. Divide each group by (A) younger‐unfit group, (B) elderly‐fit group, and (C) frail group, and then analyzed overall survival (OS) and progression‐free survival (PFS) respectively according to ibrutinib‐responsiveness

Considering that the treatment options might change depending on the patient's age and conditions and the administration stages of ibrutinib could be different, subgroup analysis was performed by younger‐unfit (n = 10), elderly‐fit (n = 14), and frail patient group (n = 9). In younger‐unfit and frail group, there were no survival differences between ibrutinib‐responder and ibrutinib‐failure statistically (*P* = .300 of OS and *P* = .115 of PFS in younger‐unfit subgroup; *P* = .747 of OS and *P* = .805 of PFS in frail subgroup, Figure 5A,C). However, in the elderly fit subgroup, OS and PFS showed a favorable result with statistical significance in ibrutinib responder (*P* = .021 of OS and *P* = .0003 of PFS, Figure 5B). Although this finding was limited with less meaningful statistical interpretation due to the very few number of patients in each subgroup, in the elderly group, because the conventional salvage chemotherapy could not be adopted to this group easily, it might be suggested that responsiveness of ibrutinib had a significant impact on survival outcomes.

Failure after ibrutinib treatment leads to a dismal prognosis; thus, effective therapy is an unmet need for these patients. Although the number of patients was very small, the current results revealed an ORR of 33% (2/6) for the first salvage chemotherapy after ibrutinib failure, with a median DOR of 3.2 months (range, 1.8‐6.1 months). These results are consistent with previous reports describing survival outcomes after ibrutinib refractoriness: an ORR of 30% (18/61) with a median OS of 2.9 months by Martin et al,^12^ and an ORR of 35% (6/17) and median OS of 4 months by Cheah et al.^25^ Several studies of large numbers of patients have revealed no uniquely successful therapies, including allo‐HSCT, in the post‐ibrutinib setting.^12^ Similarly, the current data showed that neither MIPI score nor the choice of salvage chemotherapy predicted survival after ibrutinib failure (data not shown). It is unclear why MIPI score and prior bendamustine exposure status were not statistically significant factors influencing survival in our cohort, but this may be explained by the limited number of patients in the post‐ibrutinib group. There was also limited statistical power to evaluate all possible predictors of survival, such as pre‐ibrutinib therapies. Nevertheless, although a much larger cohort and extended long‐term survival analysis are needed, the difference from previous reports is that allo‐HSCT improved survival outcomes without treatment‐related mortality after ibrutinib failure in the current study.

Currently, upfront auto‐HSCT is generally recommended for younger and fit patients in MCL treatment.^11^, ^26^ However, auto‐HSCT is often not available due to disease characteristics of MCL. Therefore, since this study included that all patients were treated with R‐CHOP as frontline chemotherapy and only six patients were treated with upfront auto‐HSCT, it is considered to be meaningful as an indirectly identifying the therapeutic response and prognosis of ibrutinib in the group of the elderly or auto‐HSCT unfit.

Our study had several limitations. First, it had a retrospective designed study, so it was possible to include biases in these data. Most of all, the smaller sample size is the main problem of this study; statistical results for each factor analysis should be interpreted with caution regarding the small number of patients. In addition, our data identified to much higher ORR than previously reported large prospective studies, it suggested that response evaluation might have differed from rigorous evaluations performed in the context of clinical trials as well as small size cohort.

To summarize the results of our analysis, we must ensure that the therapeutic effects of ibrutinib are maintained for as long as possible to improve the survival outcomes in patients with relapsed or refractory MCL. Interestingly, although the number of patients was too small to draw any definitive conclusions, prior bendamustine exposure was associated with inferior survival outcomes. This needs to warrant further verification in a larger number of patients in future studies. Moreover, allo‐HSCT is currently a possible salvage therapeutic strategy option after ibrutinib failure. A previous report recommended continuing a once‐daily dose of ibrutinib, unlike other conventional chemotherapies, until disease progression or unacceptable toxicity is noted.^7^ It is inevitable that patients will experience a loss of response while maintaining ibrutinib therapy. Therefore, inconsistent responders should have a therapeutic strategy that includes preparation for allo‐HSCT in advance.

In conclusion, the current study confirmed favorable ORR and DOR for ibrutinib‐treated patients with relapsed or refractory MCL. Unlike ibrutinib responders, ibrutinib failure patients with a high risk of biologic MIPI had very poor survival outcomes, and there was no promising salvage treatment after refractoriness to ibrutinib. We confirm again the recent observations of Martin et al^12^ and Cheah et al^25^ that ibrutinib failures have inferior survival outcomes, and these findings are not surprising or novel. Despite the potential bias of the retrospective observational study design and the limited number of patients, this study represents a real‐world population in daily clinical practice, and our research might have been meaningful in that it also raised a premature question about a sequential relationship with ibrutinib and other salvage chemotherapy such as bendamustine.

## Data Availability

The data that support the findings of this study are available on request from the corresponding author. The data are not publicly available due to privacy or ethical restrictions.
