# Supplementary material for: Clinical outcomes for ibrutinib in relapsed or refractory mantle cell lymphoma in real‐world experience
Source: Cancer Med. 2019 Sep 27;8(16):6860–70. doi: 10.1002/cam4.2565 (PMC6853811; doi:10.1002/cam4.2565)
Supplement: Supplementary file 1 [file CAM4-8-6860-s001.docx]

**Supplementary Table 1. Univariable analysis for predictive prognostic factors affecting to ibrutinib response**

| Factors | OS | | PFS | | |
| --- | --- | --- | --- | --- | --- |
|  | HR (95% CI) | *p-value* |  | HR (95% CI) | *p-value* |
| Age at diagnosis (year) by median  < 64  ≥ 64 | 1  2.71 | 0.78-9.47  0.118 |  | 1  2.08 | 0.73-5.94  0.171 |
| Gender  male  female | 1  0.001 | 0-0.0001  0.990 |  | 1  0.337 | 0.043-2.61  0.298 |
| ECOG performance status at diagnosis |  |  |  |  |  |
| 0-1 | 1 | 0.71-10.36 |  | 1 | 0.90-9.05 |
| ≥ 2 | 2.71 | 0.145 |  | 2.86 | 0.074 |
| LDH  normal  elevated | 1  3.12 | 0.94-10.29  0.062 |  | 1  2.56 | 0.92-7.12  0.071 |
| Bone marrow involvement  no  yes | 1  1.08 | 0.31-3.71  0.907 |  | 1  1.9 | 0.60-6.00  0.274 |
| Ki-67  <30%  ≥30% | 1  2.60 | 0.79-8.56  0.115 |  | 1  1.96 | 0.71-5.42  0.195 |
| Lines of previous therapy |  |  |  |  |  |
| 1 | 1 | 0.75-9.58 |  | 1 | 0.67-6.20 |
| ≥ 2 | 2.68 | 0.131 |  | 2.04 | 0.208 |
| B symptom |  |  |  |  |  |
| no | 1 | 0.41-4.48 |  | 1 | 0.48-3.67 |
| yes | 1.36 | 0.612 |  | 1.33 | 0.584 |
| GI tract involvement |  |  |  |  |  |
| no | 1 | 0.43-4.85 |  | 1 | 0.72-5.64 |
| yes | 1.44 | 0.557 |  | 2.01 | 0.184 |
| IPI classification  low to low-int.  high-int. to high | 1  1 | 1-1  0.014 |  | 1  1 | 1-1  0.003 |
| standard MIPI  low to int.  high | 1  3.51 | 1.06-11.69  0.041 |  | 1  2.73 | 0.95-7.84  0.061 |
| simplified MIPI  low to int.  high | 1  2.62 | 0.76-9.00  0.128 |  | 1  2.26 | 0.76-6.70  0.140 |
| biologic MIPI  low to int.  high | 1  15.13 | 1.92-119.3  0.009 |  | 1  4.18 | 1.31-13.37  0.016 |
| combined MIPI  low to low-int.  high-int. to high | 1  5.64 | 1.62-19.56  0.006 |  | 1  3.68 | 1.31-10.31  0.013 |
| Prior auto-HSCT  no  yes | 1  1.36 | 0.36-5.18  0.653 |  | 1  0.99 | 0.28-3.53  0.988 |
| Allo-HSCT after ibrutinib therapy  no  yes | 1  0.83 | 0.11-6.84  0.865 |  | 1  2.33 | 0.63-8.63  0.207 |
| Prior bendamustine-based therapy  no  yes | 1  1.64 | 0.73-3.67  0.228 |  | 1  2.507 | 0.75-8.41  0.048 |
| Bendamustine after ibrutinib therapy |  |  |  |  |  |
| no | 1 | 0.24-1.26 |  | 1 | 0.06-2.00 |
| yes | 0.55 | 0.157 |  | 0.35 | 0.239 |
| Response to ibrutinib at first 3 cycles  Sensitive (CR or PR)  Refractory (SD or PD) | 1  8.50 | 1.86-38.84  0.006 |  | 1  8.25 | 4.70-32.91  0.001 |
| Response to ibrutinib at ongoing (overall)  Sensitive (CR or PR)  Refractory (SD or PD) | 1  4.93 | 1.29-18.79  0.02 |  | - | - |
